# Association between IL-32 genotypes and outcome in infection-associated acute lung injury

**DOI:** 10.1186/cc10258

**Published:** 2011-06-07

**Authors:** John J Arcaroli, Nianjun Liu, Nengjun Yi, Edward Abraham

**Affiliations:** 1 Department of Medicine, University of Colorado Anschutz Medical Campus, 12801 E. 17th Avenue, Aurora, CO 80045, USA; 2 Department of Biostatistics, Section on Statistical Genetics, University of Alabama at Birmingham, 1530 3rd Avenue South, Birmingham, AL 35294-0012, USA; 3 Department of Medicine, University of Alabama at Birmingham, 1530 3rd Avenue South, BDB 420, Birmingham, AL 35294-0012, USA

## Abstract

**Introduction:**

Our purpose was to investigate variation within the IL-32 promoter and gene, and susceptibility to and outcomes from infection associated acute lung injury (ALI).

**Methods:**

Retrospective case-control study involving healthy individuals (controls) and patients (cases) with infection-associated ALI. Two hundred fifty-eight healthy normal controls and 251 patients with infection-associated ALI were used for comparison. The IL-32 promoter/gene was sequenced in 52 healthy Caucasian individuals to identify single nucleotide polymorphisms (SNPs). Allelic discrimination was performed on 11 SNPs to determine differences between cases and controls and outcomes in patients with infection associated ALI.

**Results:**

Logistic and normal regression models were used to evaluate the associations with SNPs in cases and controls, and outcomes in patients with infection associated ALI. rs12934561, an intronic SNP, was found to be associated with risk for ALI in the case-control study and with more severe clinical course, as shown by increased time on the ventilator and the presence of fluid unresponsive hypotension. Further, it was found that rs12934561 has gender-specific effects and strongly interacts with other SNPs.

**Conclusions:**

A common IL-32 genotype, rs12934561, is associated with the risk of ALI as well as the need for prolonged mechanical ventilatory support. This finding suggests that IL-32 is not only involved in the initiating inflammatory and cellular events that result in ALI, but also participates in determining the severity of pulmonary dysfunction associated with ALI.

## Introduction

Acute lung injury (ALI) and its more severe form, the acute respiratory distress syndrome (ARDS), are frequently associated with sepsis [1,2]. Major cellular populations involved in these processes include alveolar and tissue-based macrophages, epithelial and endothelial cells, and activated neutrophils that migrate from the circulation into the interstitial and alveolar spaces, where they release reactive oxygen species and proinflammatory cytokines and chemokines, including TNF-α, IL-1β, and IL-8 [3-8]. The acute inflammatory process associated with ALI disrupts epithelial and endothelial integrity, resulting in interstitial edema, leakage of protein into the alveolar space, and tissue injury [9].

IL-32 has been proposed to play an important role in modulating innate and adaptive responses to infection. In initial studies, IL-32 was shown to induce the expression of TNF-α and IL-8 via the activation of p38 and nuclear factor-kappa B (NF-κB) [10]. IL-32 has also been shown to associate with nucleotide oligomerization domain (NOD) 1 and NOD2 ligands, facilitating the release of IL-1β and IL-6 in a manner independent of Toll-like receptor (TLR) activation [11]. IL-32 has been reported to be constitutively expressed in human umbilical vein endothelial cells and pulmonary vascular endothelial cells, suggesting a role for IL-32 in endothelial inflammation and endothelial initiated events, including intravascular coagulation [12]. Increased levels of IL-32 are present in the lungs of patients with chronic obstructive pulmonary disease and correlate with airflow decreases that are associated with this condition [13]. Additionally, IL-32 has been linked to other inflammatory diseases, including psoriasis [14,15], Crohn disease [16], and rheumatoid arthritis [14-19].

The *IL-32 *gene is located on chromosome 16 p13.3 and comprises four splice variants: IL-32 α, β, δ, and γ [10]. A study by Choi and colleagues [20] demonstrated that, although all four isoforms are biologically active, IL-32γ was the most potent at inducing the production of the cytokines in peripheral blood mononuclear cells and macrophages. Polymorphisms have been described in the *IL-32 *gene by the National Center for Biotechnology Information; however, association studies between IL-32 polymorphisms and disease have not been reported. Therefore, given the involvement of IL-32 in inflammation and its association with diseases associated with dysregulated inflammatory responses, we hypothesized that single-nucleotide polymorphisms (SNPs) within the *IL-32 *gene or promoter (or both) would be associated with susceptibility to or outcomes in patients with infection-associated ALI.

## Materials and methods

### Case control

#### Volunteer selection

Healthy Caucasian volunteers, 19 to 89 years of age, were recruited to determine polymorphisms in the *IL-32 *gene. The volunteer population consisted of 115 males (with a mean age of 32.0 ± 8.9 years) and 143 females (with a mean age of 37.0 ± 11.6 years). Each volunteer signed an informed consent document. The study was approved by the University of Colorado institutional review board.

#### Patients with acute lung injury

All patients included in this study had been enrolled in National Institutes of Health (NIH) ARDS Network studies and had received low tidal volume ventilation in accordance with previously published protocols and fulfilled all NIH ARDS Network protocol inclusion and exclusion criteria [21]. All patients were Caucasian and had infection-associated ALI as characterized by either systemic or localized pulmonary infection as the investigator-identified primary etiology for ALI. ALI was defined by standard criteria [21]. Septic shock was defined as a systolic blood pressure of less than 90 mm Hg for at least 30 minutes despite fluid replacement or the use of inotropic support to maintain blood pressure. This genetic analysis study was approved by the University of Colorado Health Sciences Center and the University of Alabama at Birmingham institutional review boards. Consent was obtained from all patients or their surrogates before enrollment as part of their enrollment into the NIH protocols.

#### Statistical analysis

##### Multiple-SNP logistic and linear models

We used two logistic and linear regression models to analyze our data. The first model (the main-effect model) simultaneously fitted age and sex, and all 22 main effects (that is, both additive and dominant effects) of the 11 SNPs. The second model (the interacting model) simultaneously fitted age, sex, 22 main effects, 22 SNP-sex interactions, and 220 epistatic interactions.

We standardized the continuous covariate, age, to have a mean of 0 and a standard deviation of 0.5, and coded sex as 0/1 binary variables, indicating males/females, respectively. For each SNP, we denoted common homozygote (that is, the homozygote with higher frequency), heterozygote, and rare homozygote by c, h, and r, respectively. We coded the main-effect predictors by using the Cockerham genetic model. The Cockerham model defines two main effects for each SNP (that is, additive and dominance effects, denoted by the suffixes 'a' and 'd' at the end of the SNP rs numbers). The additive predictor is coded as -1, 0, and 1 for c, h, and r, and the dominance predictor as -0.5 for c and r and 0.5 for h, respectively [22,23]. The additive effect 'a' represents the genotypic effect (r - c)/2, and the dominance effect 'd' measures h - (c + r)/2. A positive additive effect (for example, odds ratio (OR) >1) indicates that the rare homozygote increases risk compared with the common homozygote, and a positive dominance effect means that the heterozygote increases risk compared with the mean of two homozygotes. The SNP-sex and epistatic predictors were constructed by multiplying two corresponding main-effect variables, introducing two SNP-sex interactions for each SNP (that is, sex-additive and sex-dominance interactions) and four epistatic interactions for a pair of SNPs (that is, additive-additive, additive-dominance, dominance-additive, and dominance-dominance interactions).

Multiple-SNP analysis relieves the problem of multiple testing encountered in single-SNP analysis, can accommodate linkage disequilibrium (LD) among the SNPs, and has the advantage of providing potentially increased power and reduced false-positives to detect causal variants, better separating highly correlated predictors, and more efficiently detecting epistatic interactions [24-26]. However, traditional regression procedures are usually problematic, owing to potentially large numbers of correlated and complex variables. We addressed such issues by using a Bayesian approach that places appropriate prior distributions on coefficients to obtain stable estimates. Specifically, we used Student-t prior distributions, i.e.,and, with the hyperparameters (*v*_*j*_,*s*_*j*_) reasonably preset, where *βj *is the *j*^th ^coefficient in the model [25,26]. For covariates and main effects of SNPs, we used the weakly informative priors by setting (*v*_*j*_,*s*_*j*_) to be (1, 2.5) [27]. For sex-gene and epistatic interactions, we chose (1, 2.5 K_main_/K_GE_) and (1, 2.5K_main_/K_GG_), respectively, where K_main_, K_GE_, and K_GG _are the total numbers of main, SNP-sex, and epistatic effects in the model [26]. We used the expectation-maximization algorithm to fit the models with the Student *t *priors by estimating the marginal posterior modes of the coefficients [25,26].

We used two summary measures, the deviance and the Akaike information criterion (AIC), to compare different models. Lower deviance and AIC mean better fit to data. On the basis of the fitted multiple-SNP logistic models, we calculated the average predictive probability for each genotype of each SNP averaging over all the data points in the dataset and all other variables. We performed similar calculations for each pair of SNPs that showed significant epistatic interactions. We also calculated the gender-specific average predictive probabilities for SNPs that showed significant sex-specific effects. The average predictive probabilities show which genotypes increase or are protective against the risk and how a genotype depends on the genotypes of another SNP or covariate.

##### Haplotype analysis

We performed haplotype analysis on the data as a secondary approach complementary to the above analysis. We employed the haplotype-sharing approach, which is implemented in the software CHASe [28]. Because CHASe handles genotype data only, age and sex were not included in the haplotype analysis.

## Results

### Sequencing of the *IL-32 *gene

Sequencing the *IL-32 *gene and promoter identified a total of 23 SNPs with allele frequencies ranging from 6% to 48% (Additional file 1, Table S1). Nine SNPs were in the promoter and 14 were located within the gene. One SNP in the promoter (-954) and one in the intron (613) had not been previously described. Rs4786370, rs55699988, and rs28372698 (*r*^*2 *^= 0.87) as well as rs56037566 and rs11861531 (*r*^*2 *^= 0.84) were tag SNPs. Analysis of pairwise LD was relatively weak for the IL-32 promoter and gene within this population (Additional file 1, Figure S1).

### Characteristics of the ARDS Network population

Two hundred fifty-one Caucasian patients who had infection-induced ALI and who were enrolled from NIH ARDS Network trials were available for analysis. As shown in Table 1, the average age of patients was 51.3 ± 15.6 years, and men and women were equally represented. Overall mortality for this population was 25%. The primary source of infection was the lung, and pulmonary infections were present in 55% of the patients. An additional 258 healthy Caucasians were used to determine associations between cases and controls.

**Table 1:** Characteristics of acute lung injury in the ARDS Network patient population

Number	251
Age in years	
Mean ± SD	51.3 ± 15.6
Range	17 to 90
Male	50.0%
APACHE III, mean ± SD	96.2 ± 29.7
Mechanical ventilation	100%
Mortality	
Overall	25.0%
Male	26.0%
Female	24.0%
Source of infection	
Lung	55.0%
Peritoneum	4.0%
Vascular line	4.0%
Skin	2.0%
GI/Biliary tract	2.0%
Other	33.0%

APACHE III, Acute Physiology and Chronic Health Evaluation III; ARDS, acute respiratory distress syndrome; GI, gastrointestinal; SD, standard deviation.

### Case-control study

**Figure 1:**
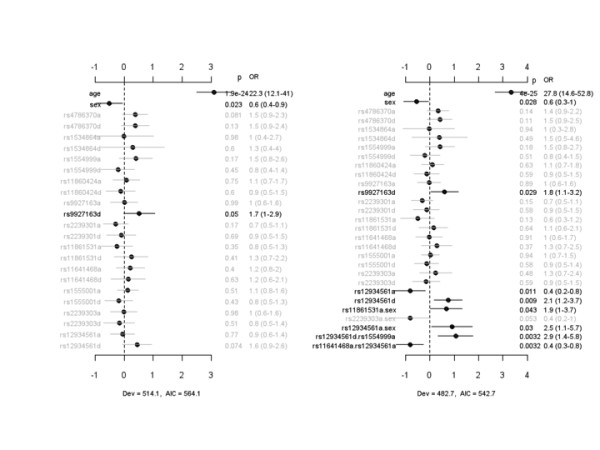
**Case-control study of the association of IL-32 SNPs with risk for developing infection-associated acute lung injury**. The left panel represents the analysis of simultaneously fitting age, sex, and main effects for all 11 identified IL-32 SNPs. The right panel represents the analysis of simultaneously fitting age, sex, and main effects of all SNP, sex-gene, and epistatic interactions. The notation for main effects, a and d, indicates additive and dominance effects, respectively. The term X_1_.X_2 _represents interaction between X_1 _and X_2_. Interactions with a *P *value of greater than 0.05 are not displayed. The points and short lines represent estimates of effect and ± 2 standard errors, respectively. The numbers at the right side are the *P *values (p), odds ratios (ORs), and 95% confidence intervals. The deviance (Dev) and Akaike information criterion (AIC) under each model are also shown. IL, interleukin; SNP, single-nucleotide polymorphism.

Prior to the determination of whether IL-32 SNPs were associated with risk for ALI, Hardy-Weinberg equilibrium (HWE) was evaluated on cases and controls. SNPs rs11860424 and rs9927163 showed some departure from HWE (*P *< 0.05). Next, we compared 11 out of the 23 SNPs (which captured 14 out of the 23 SNPs using tag SNPs) between healthy controls and patients with infection-associated ALI. We attempted to analyze the other SNPs identified by sequencing; however, TaqMan assays for these SNPs failed. Figure 1 displays the coefficient estimates, standard errors, *P *values, and ORs for all of the effects under the main-effect and interacting models for the case-control study. The main-effect model detected rs9927163 with a marginally significant dominance effect, which was also present in the interacting model. The interaction analysis identified two sex-specific effects, rs11861531a.sex and rs12934561a.sex, and two epistatic interactions, rs12934561d.rs1554999a and rs11641468a.rs12934561a. The association of rs12934561 with risk for ALI, which was not significant under the main-effect model, became significant under the interacting model. The interacting model had lower deviance and AIC than the main-effect model, indicating that inclusion of sex-specific effects and epistatic interactions improved the fit of the model to data.

For rs9927163, the heterozygote increased the risk of developing ALI whereas homozygotes decreased the risk (Additional file 1, Figure S2). The genotypic effects of rs11861531 and rs12934561 depended on gender, with males demonstrating relatively greater protection from ALI than females, and the genotypic effects of the interacting SNP varied with the genotypes of the second SNP.

### Analysis of IL-32 genotypes and death associated with acute lung injury

**Figure 2:**
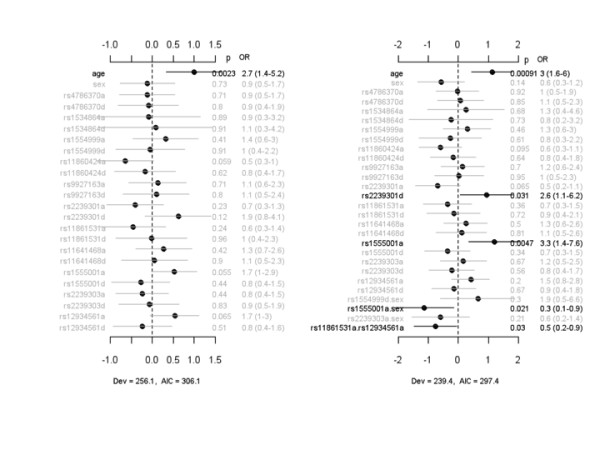
**Association between IL-32 SNPs and 60-day mortality in patients with infection-associated acute lung injury**. The left panel represents the analysis of simultaneously fitting age, sex, and main effects of all the SNPs. The right panel represents the analysis of simultaneously fitting age, sex, and main effects of all the SNP, sex-gene, and epistatic interactions. The notation for main effects, a and d, indicates additive and dominance effects, respectively. The term X_1_.X_2 _represents interaction between X_1 _and X_2_. Interactions with a *P *value of greater than 0.05 are not displayed. The points and short lines represent estimates of effects and ± 2 standard errors, respectively. The numbers at the right side are the *P *values (p), odds ratios (ORs), and 95% confidence intervals. The deviance (Dev) and Akaike information criterion (AIC) under each model are shown. IL, interleukin; SNP, single-nucleotide polymorphism.

Figure 2 displays the coefficient estimates, standard errors, *P *values, and ORs for the main-effect model and the interacting model for death at 60 days (that is, death or not by day 60) after study inclusion. The main-effect model detected no SNP that was significantly associated with death at day 60. However, the interaction analysis found that rs2239301d and rs1555001a were associated with an increased risk of death in patients with infection-associated ALI. There were also a sex-specific effect, rs1555001a.sex, and one epistatic interaction, rs11861531a.rs1293461a, that were both associated with diminished risk for death at day 60. The interacting model had lower deviance and AIC than the main-effect model, indicating that inclusion of sex-specific effects and epistatic interactions improved the fit of the model to data (Additional file 1, Figure S3). The gene-sex interaction of rs1555001 occurred mainly for the rare homozygote, with increased risk of death among women with this SNP, and the gene-gene interaction of rs11861531 and rs1293461 occurred mainly for the common homozygote of rs11861531 and rare homozygote of rs1293461 (Additional file 1, Figure S3).

### IL-32 SNPs and time on the ventilator and presence of shock among patients with acute lung injury

**Figure 3:**
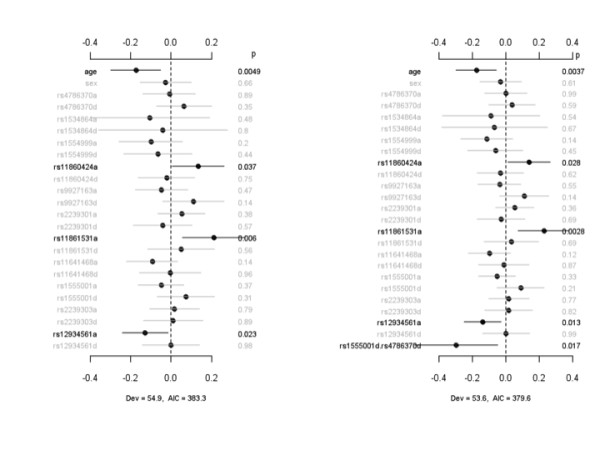
**Association between IL-32 SNPs and ventilator-free days**. The left panel represents the analysis of simultaneously fitting age, sex, and main effects of all the SNPs. The right panel represents the analysis of simultaneously fitting age, sex, and main effects for all the SNP, sex-gene, and epistatic interactions. The notation for main effects, a and d, indicates additive and dominance effects, respectively. The term X_1_.X_2 _represents interaction between X_1 _and X_2_. Interactions with a *P *value of greater than 0.05 are not displayed. The points and short lines represent estimates of effects and ± 2 standard errors, respectively. The numbers at the right side are the *P *values (p). The deviance (Dev) and Akaike information criterion (AIC) under each model are shown. IL, interleukin; SNP, single-nucleotide polymorphism.

**Figure 4:**
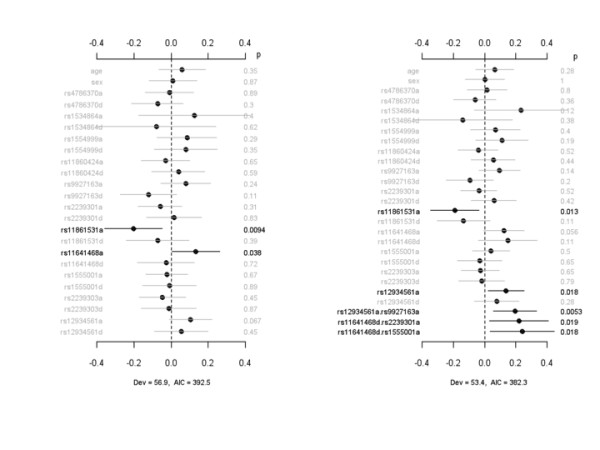
**Association between IL-32 SNPs and days on ventilator**. The left panel represents the analysis of simultaneously fitting age, sex, and main effects of all the SNPs. The right panel represents the analysis of simultaneously fitting age, sex, and main effects of all the SNP, sex-gene, and epistatic interactions. The notation for main effects, a and d, indicates additive and dominance effects, respectively. The term X_1_.X_2 _represents interaction between X_1 _and X_2_. Interactions with a *P *value of greater than 0.05 are not displayed. The points and short lines represent estimates of effects and ± 2 standard errors, respectively. The numbers at the right side are the *P *values (p). The deviance (Dev) and Akaike information criterion (AIC) under each model are shown. IL, interleukin; SNP, single-nucleotide polymorphism.

To investigate a possible association between severity of lung injury and SNPs, we analyzed the number of ventilator-free days (VFDs) associated with each IL-32 SNP for patients with infection-associated ALI. Figure 3 displays the coefficient estimates, standard errors, and *P *values for the main-effect model and the interacting model for VFDs, an indicator of need for mechanical ventilation. The main-effect model detected three SNPs - rs11860424, rs11861531, and rs12934561 - significantly associated with VFDs through additive effects. The interacting model identified not only these three additive effects but also an additional epistatic effect, rs1555001d.rs4786370d, that was associated with reduced VFDs. The interacting model reduced deviance and AIC and thus improved the model fit to the data. In a second analysis (Figure 4), we evaluated the association between IL-32 SNPs and days on the ventilator (DOVs), another indicator of the need for mechanical ventilation in critically ill patients with respiratory failure. The main-effect model detected two SNPs, rs11861531a and rs11641468a, that were significantly associated with DOVs. rs11861531a was associated with a reduction in the duration of requirement for mechanical ventilation, whereas the time on mechanical ventilation was significantly greater among patients with rs11641468. Under the interacting model, the effect for rs11861531a, but not that for rs11641468a, was still significant. The interaction analysis identified an additional SNP, rs12934561a, that was associated with an increased number of DOVs and three epistatic interactions, rs12934561a.rs9927163a, rs11641468d.rs2239301a, and rs11641468d.rs1555001a.

**Figure 5:**
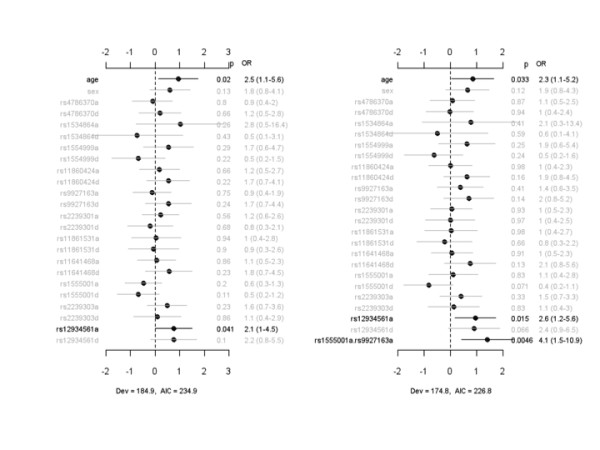
**Association between IL-32 SNPs and shock, using the Brussels score criteria for vasopressor requiring hypotension**. The left panel represents the analysis of simultaneously fitting age, sex, and main effects of all the SNPs. The right panel represents the analysis of simultaneously fitting age, sex, and main effects of all the SNP, sex-gene, and epistatic interactions. The notation for main effects, a and d, indicates additive and dominance effects, respectively. The term X_1_.X_2 _represents interaction between X_1 _and X_2_. Interactions with a *P *value of greater than 0.05 are not displayed. The points and short lines represent estimates of effects and ± 2 standard errors, respectively. The numbers at the right side are the *P *values (p), odds ratios (OR), and 95% confidence intervals. The deviance (Dev) and Akaike information criterion (AIC) under each model are shown. IL, interleukin; SNP, single-nucleotide polymorphism.

We next examined whether particular IL-32 SNPs were associated with hemodynamic shock as assessed by the Brussels criteria requirement of vasopressor support to maintain normotension or by a systolic blood pressure of less than 90 mm Hg despite adequate fluid administration. Figure 5 shows the coefficient estimates, standard errors, and *P *values for all of the effects under the main-effect model and the interacting model for shock using the Brussels score values. The main-effect model detected one SNP, rs12934561a, significantly associated with shock. The interacting model identified not only this main effect but also one epistatic interaction, rs1555001a.rs9927163a. This interaction reduced deviance and AIC and thus improved the model fit to the data. For rs1293461, the common homozygote was associated with decreased risk of developing shock in patients with infection-associated ALI, and the effects of rs1555001 varied with the genotypes of rs9927163 (Additional file 1, Figure S4).

The main-effect model detected three SNPs - rs11641468d, rs2239303d, and rs12934561d - that were significantly associated with shock, as defined by sequential measurements of vital signs (Additional file 1, Figure S5). The interacting model identified not only the same three SNPs but also one epistatic interaction (that is, rs11641468a.rs2239301a) (Additional file 1, Figure S6). The genotypic effects of rs11641468 varied with the genotypes of rs2239301 (Additional file 1, Figure S6).

### Haplotype analysis

Because the haplotype method handles genotype data only, the results are unadjusted for any covariates. Table S2 of Additional file 1 shows the haplotypes and their corresponding frequencies. The smallest *P *values from analyzing each of the aforementioned phenotypes are all within the region 1474 to 4153 (consisting of SNPs rs9927163, rs2239301, rs11861531, rs11641468, rs1555001, rs2239303, and rs12934561). After Bonferroni correction, only the case-control and shock (as defined by Brussels score criteria) phenotypes showed significant signals. For the case-control phenotype, the smallest *P *value is for the region 1544 to 2642 (consisting of SNPs rs2239301, rs11861531, rs11641468, and rs1555001; *P *value = 0.036 after Bonferroni correction). However, several regions between 1474 and 4153 have significant *P *values even after Bonferroni correction. For shock defined by Brussels score criteria, the smallest *P *value is in the region 3225 to 4153 (consisting of SNPs rs2239303 and rs12934561; *P *value = 0.008 after Bonferroni correction). The region 2642 to 4153 (consisting of SNPs rs1555001, rs2239303, and rs12934561) is significant as well (*P *value = 0.029 after Bonferroni correction).

## Discussion

IL-32 is a recently described cytokine that demonstrates increased expression after engagement of TLRs and IL-1 receptors in endothelial, epithelial, monocytic, and other cell populations and that induces the production of proinflammatory cytokines, including TNF-α and IL-1β, through intracellular pathways involving NF-κB and the p38 MAP kinase [16,29-31]. IL-32 appears to play an important role in pathophysiologic states associated with dysregulated inflammatory responses, including rheumatoid arthritis, chronic obstructive pulmonary disease, psoriasis, and inflammatory bowel disease [13,18,32]. In a mouse model of sepsis, IL-32 was shown to potentiate peritonitis-induced elevations in serum levels of TNF-α and IL-1β, and overexpression of the IL-32β isoform led to reduced time to death [33].

ALI is accompanied by an intense inflammatory response in the lungs, driven by the proinflammatory cytokine and chemokine generation that initiates and accompanies the accumulation of large numbers of activated neutrophils in the pulmonary interstitium and airways [34]. ALI is also characterized by endothelial alterations that are associated with microvascular abnormalities and epithelial dysfunction that leads to the leakage of proteins into the alveolar space, resulting in diminished lung compliance and hypoxemia [35,36]. While there is limited information linking IL-32 to ALI, involvement of this cytokine in the inflammatory reaction that characterizes ALI, as suggested by the present data, is not surprising, especially given that sepsis associated with pulmonary or extra-pulmonary infections is the most common etiologic factor for ALI [35,36]. However, previous studies have not specifically examined either the role of IL-32 in ALI or the possibility that genetically determined alterations in IL-32 might be associated with risk for or outcome from ALI.

In the present studies, we characterized 23 SNPs in the IL-32 promoter and gene. One SNP (-954) in the promoter and an intronic SNP (613) had not previously been described. When the IL-32 genotypes were examined in a case-control study, two intronic SNPs were associated with enhanced risk for developing ALI. In particular, heterozygosity of rs9927163 was associated with an increased likelihood for the development of ALI, whereas rs12934561 was associated with risk for ALI mainly through sex-specific effects and epistatic interactions, and a diminished effect was found primarily in men. Both of these genotypes are fairly common in Caucasian individuals; the allele frequency for rs22967163 is approximately 25% and that for rs12934561 is about 41%. While the functional significance of these genotypes in terms of IL-32 expression is unknown at present, these findings indicate that IL-32 genotypes are associated with risk for development of ALI and suggest that IL-32 occupies an important role in the pathogenesis of ALI.

Several IL-32 SNPs were significantly associated with clinical outcome among patients with ALI. In particular, rs12934561, an intronic SNP that was found to be associated with risk for ALI in the case-control study, also demonstrated an association with a more severe clinical course, as defined by a longer period on the ventilator, using both VFDs and time on ventilator as measures of the need for mechanical ventilation. rs12934561 was also associated with a higher prevalence of fluid unresponsive hypotension. Although effects of other SNPs, including rs223930d, rs1555001a, rs11641468d, and rs2239303d, were associated with worse clinical outcome (such as increased mortality at day 60, prolonged requirement for mechanical ventilation, or the presence of shock), none of these genotypes was present for multiple clinical parameters, as was the case of rs12934561.

The present results indicate that IL-32 genotypes are associated not only with risk for the development of ALI but also with outcome from this critical illness. Such associations between IL-32 SNPs and ALI are not surprising given the multiple cellular populations that express this cytokine and that participate in the pathogenesis of ALI, including macrophages, endothelial, and epithelial cells, as well as the ability of IL-32 to affect inflammatory and other pathways known to mediate pulmonary and extra-pulmonary organ dysfunction occurring with ALI. In particular, IL-32 expression is enhanced after engagement of TLR4 receptors by ligands, including LPS, that play a major initiating role in ALI due to sepsis, hemorrhage, trauma, or hyperoxia [11,12,32,33]. IL-1β and TNF-α, two cytokines whose expression is induced by IL-32, have been shown to be present in high concentrations in the lungs during ALI and to participate in potentiating neutrophil recruitment and producing pulmonary injury in this setting [8,37,38]. Of note, IL-1β, which is found in high concentrations in the alveolar space and airways during ALI, induces IL-32 expression and thereby may participate in potentiating inflammatory cascades, resulting in upregulation of its own levels as well as those for IL-32 [12,32,33]. IL-32 has been shown to upregulate the C-X-C chemokine IL-8 [12], which is known to be expressed at high levels in the lungs during ALI and has potent ability to enhance recruitment of neutrophils, a cell population that plays a major role in mediating pulmonary injury during ALI, into the pulmonary parenchyma and airways [39-41].

Although IL-32 SNP rs12934561 was shown to be associated with risk for and worse outcomes from ALI, there are several limitations with this study. Only 11 out of the 23 SNPs were genotyped (12 failed assay design), and LD between rs12934561 and one or more of these SNPs could possibly exist. Therefore, the potential SNP associated with outcome from ALI may not be rs12934561. In addition, the sample size in this study is not robust. Therefore, it is possible that the association between ALI and other SNPs may be missed because of limited statistical power. Although our analysis controls type I error rate well, there is the possibility that rs12934561 is a false-positive finding, and this finding will require confirmation with a validation population, preferably with larger sample size and denser markers. Finally, functional studies of rs12934561 were not performed; thus, further studies are warranted to have a better understanding of the role of this IL-32 SNP in altering IL-32 function and ultimately outcomes in ALI.

## Conclusions

While the present studies do not establish the functional significance of the IL-32 SNPs found to be associated with risk for and outcome from ALI, they do support the hypothesis that IL-32 plays an important role in the pathogenesis of ALI. The fact that a common IL-32 genotype, rs12934561, was associated with ALI and interacted with other SNPs as well as the need for prolonged mechanical ventilatory support in patients with this condition suggests that IL-32 not only is involved in the initiating inflammatory and cellular events that result in ALI but also participates in determining the severity of pulmonary dysfunction associated with ALI. Although future preclinical and clinical interventional studies will be necessary to establish the involvement of IL-32 in ALI, the present results are fully supportive of a major role for IL-32 in determining both propensity for and severity of ALI.

## Key messages

• IL-32 appears to play an important role in pathophysiologic states associated with dysregulated inflammatory responses.

• The IL-32 SNP rs12934561 is associated with an increased risk of acute lung injury (ALI) as well as the need for prolonged mechanical ventilatory support.

• These results suggest that IL-32 not only is involved in the initiating inflammatory and cellular events that result in ALI but also participates in determining the severity of pulmonary dysfunction associated with ALI.

## Abbreviations

AIC: Akaike information criterion; ALI: acute lung injury; ARDS: acute respiratory distress syndrome; DOV: day on ventilator; HWE: Hardy-Weinberg equilibrium; IL: interleukin; LD: linkage disequilibrium; NF-κB: nuclear factor-kappa B; NIH: National Institutes of Health; NOD: nucleotide oligomerization domain; OR: odds ratio; SNP: single-nucleotide polymorphism; TLR: Toll-like receptor; TNF-α: tumor necrosis factor-alpha; VFD: ventilator-free day.

## Competing interests

The authors declare that they have no competing interests.

## Authors' contributions

JJA participated in study design, sequencing, SNP genotyping, data collection, and manuscript preparation. NL and NY performed the statistical analysis and prepared the manuscript. EA participated in study design and data analysis and drafted the manuscript. All authors read and approved the final manuscript.

## Supplementary Material

Additional File 1 **Supplemental Data **Supplemental Methods and Results. Click here for file
