# Supplementary material for: Association between IL-32 genotypes and outcome in infection-associated acute lung injury
Source: Crit Care. 2011 Jun 7;15(3):R138. doi: 10.1186/cc10258 (PMC3219007; doi:10.1186/cc10258)
Supplement: Additional File 1 — Supplemental Data Supplemental Methods and Results. [file cc10258-S1.DOC]

**Supplemental Materials and Methods**

# IL-32 Sequencing

Genomic DNA samples from 52 healthy Caucasians were randomly selected from a pool of 100 healthy controls to determine IL-32 gene variation. The study was approved by the University of Colorado institutional review board, and each volunteer signed an informed consent document. Sequencing consisted of the 2 kb promoter and gene through the 3’ untranslated region of IL-32. Genomic DNA was amplified using specific forward and reverse PCR primers for the 2 kb promoter and IL-32 gene. The 50 μl PCR reaction contained approximately 50 ng of genomic DNA, 1x PCR Buffer, 100 pM of forward and reverse primers for the promoter or forward primer and reverse primers for the IL-32 gene, 50 mM dNTPs, 100 pM MgCl_2_ and 5 units of Hi-Fidelity Taq polymerase (Applied Biosystems). The PCR cycling conditions to obtain the ~ 5 kb PCR product consisted of 96°C for 3 minutes and then 20 cycles of 30 sec each @ 94°C, 1 min. at X° (where X decreases from 65°C to 55°C by 0.5°C per cycle), followed by 15 min at 68°C extension and 15 cycles of 30 sec each @ 94°C, 1 min. at 55°C, and 15 min. at 68°C + 20 seconds per cycle. Following the PCR reaction, the PCR products were treated with 5 μl of ExoSap-IT (GE HealthCare) to remove unused primers and nucleotides. The PCR product was separated by 0.75% agarose gel-electrophoresis (stained with 0.1% ethidium bromide) in order to confirm the size and quantity of the PCR product. After gel confirmation of the PCR product, sequencing was performed with 60 ng of PCR product and 5 pmol forward and reverse primers specific for the IL-32 promoter and gene.

The sequencing reactions consisted of the following procedure: Double-stranded DNA templates were sequenced by the University of Colorado Cancer Center DNA Sequencing and Analysis Core Facility using AB Prism kits from Applied Biosystems (Foster City, CA) containing AmpliTaq DNA Polymerase FS in one of the following: BigDye Terminator Cycle Sequencing Ready Reaction kit (part number 4336776) or dGTP BigDye Terminator Cycle Sequencing Ready Reaction kit (part number 4307176). The standard cycle sequencing thermo-cycling parameters were: denaturation for 5 min at 94°C, followed by 30 cycles of denaturation at 96°C for 10 sec, annealing at 50°C for 5 sec, and extension/termination at 60°C for 4 min, followed by incubation at 4°C until the samples were processed.  The reaction products were analyzed on the ABI Prism 3730 Genetic Analyzer. DNA sequence analyses of DNA were done with the Sequencher program.

Allelic Discrimination

Real-time polymerase chain reaction (PCR) allelic discrimination assays were developed by the assay-by-design service offered by Applied Biosystems (Foster City, CA). Probe and primer combinations were designed for the following SNPs: rs4786370, rs1534864, rs1554999, rs11860424, rs9927163, rs2239301, rs11861531, rs11641468, rs1555001, rs2239303, and rs12934561 which capture most SNPs with frequency greater than 1% in the Caucasian population. Unfortunately, designed primers/probe for the other 12 SNPs identified by sequencing failed. PCR reactions were performed in a final volume of 25 µl, which consisted of 1-25 ng of DNA diluted in dH_2_O, 12.5 µl of 2x Taqman Universal PCR Master Mix and 1.25 µl of 20x TaqMan SNP genotyping Assay Mix. PCR was performed using an Applied Biosystems 7300 Real-Time PCR system.

Genotyping Errors

We used statistical methods that we had previously proposed to check our data for potential genotyping errors. There is no sign of genotyping errors in cases. However, in controls, our tests showed that SNP rs11860424 may contain errors. To address this issue, we tested the association between this SNP and the risk for ALI, and found a marginal result. Our main findings concern SNP rs12934561. Therefore genotyping error is unlikely to affect our results.

Power Analysis

We performed a power analysis for two cases: one for the case control analysis (the risk of ALI) and the other for the analysis of number of ventilator free days, which represent binary and continuous responses, respectively. We focused on SNP rs12934561, which showed association with the risk of ALI, as well as the need for prolonged mechanical ventilatory support, in our study. For the case control analysis of risk of ALI, we used the software CaTS for the power analysis. We set alpha to be 0.05 and, based on our estimation of odds ratio (0.4), the powers are 0.72, 0.97, and 1 for MAF 0.02, 0.05, and larger than 0.1, using the additive model. Actually, in our study the MAF for SNP rs12934561 is 0.41. Even for an OR 50% larger than our estimates (i.e. 0.6), the powers are 0.25, 0.51, 0.75, 0.9, and 0.93 for MAF 0.02, 0.05, 0.1, 0.2, and 0.41. For ventilator free days, our estimated heritability is 0.0198, for alpha = 0.05, and the power is 0.62 for an additive effect. In addition, our power calculation is based on a single SNP. However, in our primary analysis, we analyzed all the SNPs in the same model simultaneously. This approach boosts the power further.

APACHE III Correlation

The Pearson correlation coefficient between APACHE III score and VFD is -0.4155295, with p-value 1.092e-11. The Pearson correlation coefficient between APACHE III score and TOV is 0.04228815, with p-value 0.5091.

**Supplemental Figure Legends**

**Supplemental Figure 1. Linkage disequilbruim (LD) of IL-32 in the Caucasian population.** D’ pairwise LD plot of 52 healthy Caucasians. Red: D’ =1 (LOD ≥ 2.0); blue: D’ = 1 (LOD < 2.0); Pink: D’ <1 (LOD ≥ 2.0); white: D’ <1 (LOD < 2.0).

**Supplemental Figure 2.** **Case-control study for the association of IL-32 SNPs with risk for developing infection associated ALI.** Average predictive probability for each SNP genotype, each combination of sex and SNP, and each two-locus genotype at SNPs that show significant interactions. The genotypes c, h, and r represent common homozygote, heterozygote, and rare homozygote, respectively. The notation M and F represent male and female, respectively. The dotted gray line represents the mean of probabilities.

**Supplemental Figure 3. Association between IL-32 SNPs and 60 day mortality in patients with infection associated ALI.** Average predictive probability for each SNP genotype, each combination of sex and SNP, and each two-locus genotype at SNPs that show significant interactions. The genotypes c, h, and r represent common homozygote, heterozygote, and rare homozygote, respectively. The notation M and F represents male and female, respectively. The dotted gray line represents the mean of probabilities.

**Supplemental Figure 4. Association between IL-32 SNPS and shock, using the Brussels score criteria for vasopressor requiring hypotension.** The left panel represents the analysis of simultaneously fitting age, sex, and main effects of all the SNPs. The right panel represents the analysis of simultaneously fitting age, sex, and main effects of all the SNPs, sex-gene and epistatic interactions. The notation for main effects, a and d, indicates additive and dominance effects, respectively. The term X_1_.X_2_ represents interaction between X_1_ and X_2_. Interactions with p-value > 0.05 are not displayed. The points and short lines represent estimates of effects and ± 2 standard errors, respectively. The numbers at the right side are the p-values (p), odds ratios (OR) and 95% confidence intervals. The deviance (Dev) and Akaike information criterion (AIC) under each model are also shown.

**Supplemental Figure 5. Association between IL-32 SNPs and fluid unresponsive shock, as determined by sequential vital sign measurements.** The left panel represents the analysis of simultaneously fitting age, sex, and main effects of all the SNPs. The right panel represents the analysis of simultaneously fitting age, sex, and main effects of all the SNPs, sex-gene and epistatic interactions. The notation for main effects, a and d, indicates additive and dominance effects, respectively. The term X_1_.X_2_ represents interaction between X_1_ and X_2_. Interactions with p-value > 0.05 are not displayed. The points and short lines represent estimates of effects and ± 2 standard errors, respectively. The numbers at the right side are the p-values (p), odds ratios (OR) and 95% confidence intervals. The deviance (Dev) and Akaike information criterion (AIC) under each model are also shown.

**Supplemental Figure 6. Association between IL-32 SNPs and fluid unresponsive shock, as determined by sequential vital sign measurements.** The left panel represents the analysis of simultaneously fitting age, sex, and main effects of all the SNPs. The right panel represents the analysis of simultaneously fitting age, sex, and main effects of all the SNPs, sex-gene and epistatic interactions. The notation for main effects, a and d, indicates additive and dominance effects, respectively. The term X_1_.X_2_ represents interaction between X_1_ and X_2_. Interactions with p-value > 0.05 are not displayed. The points and short lines represent estimates of effects and ± 2 standard errors, respectively. The numbers at the right side are the p-values (p), odds ratios (OR) and 95% confidence intervals. The deviance (Dev) and Akaike information criterion (AIC) under each model are also shown.

**Tables and Figures**

| **Marker** | **Position** | **Location** | **RS#** | **Base Change** | **MAF** |
| --- | --- | --- | --- | --- | --- |
| 1 | -1414 | Promoter | rs4786370 | T 🡪C | 0.314 |
| 2 | -1286 | Promoter | rs56037566 | T 🡪A | 0.147 |
| 3 | -1235 | Promoter | rs1534864 | G 🡪A | 0.098 |
| 4 | -1231 | Promoter | rs12448657 | C 🡪A | 0.441 |
| 5 | -954 | Promoter |  | G 🡪A | 0.059 |
| 6 | -920 | Promoter | rs55699988 | G 🡪C | 0.284 |
| 7 | -684 | Promoter | rs59485676 | C 🡪G | 0.157 |
| 8 | -201 | Promoter | rs28372698 | T 🡪A | 0.324 |
| 9 | -40 | Promoter | rs45499297 | T 🡪C | 0.118 |
| 10 | 613 | Intron |  | G 🡪C | 0.118 |
| 11 | 916 | Intron | rs1554999 | C 🡪A | 0.480 |
| 12 | 1458 | Intron | rs11860424 | A 🡪G | 0.304 |
| 13 | 1474 | Intron | rs9927163 | T 🡪G | 0.245 |
| 14 | 1544 | Intron | rs2239301 | T 🡪G | 0.294 |
| 15 | 1613 | Intron | rs11861531 | T 🡪C | 0.382 |
| 16 | 1821 | Intron | rs11641468 | A 🡪G | 0.127 |
| 17 | 1886 | Intron | rs72772529 | C 🡪T | 0.059 |
| 18 | 2642 | Intron | rs1555001 | T 🡪A | 0.275 |
| 19 | 3165 | Intron | rs2239302 | G 🡪A | 0.059 |
| 20 | 3225 | Intron | rs2239303 | A 🡪G | 0.314 |
| 21 | 3824 | Intron | rs2283468 | C 🡪A | 0.412 |
| 22 | 4137 | Intron | rs12922880 | T 🡪C | 0.451 |
| 23 | 4153 | Intron | rs12934561 | C 🡪T | 0.412 |

Supplemental table 1: Single nucleotide polymorphisms (SNPs) identified by sequencing the IL-32 promoter and gene.


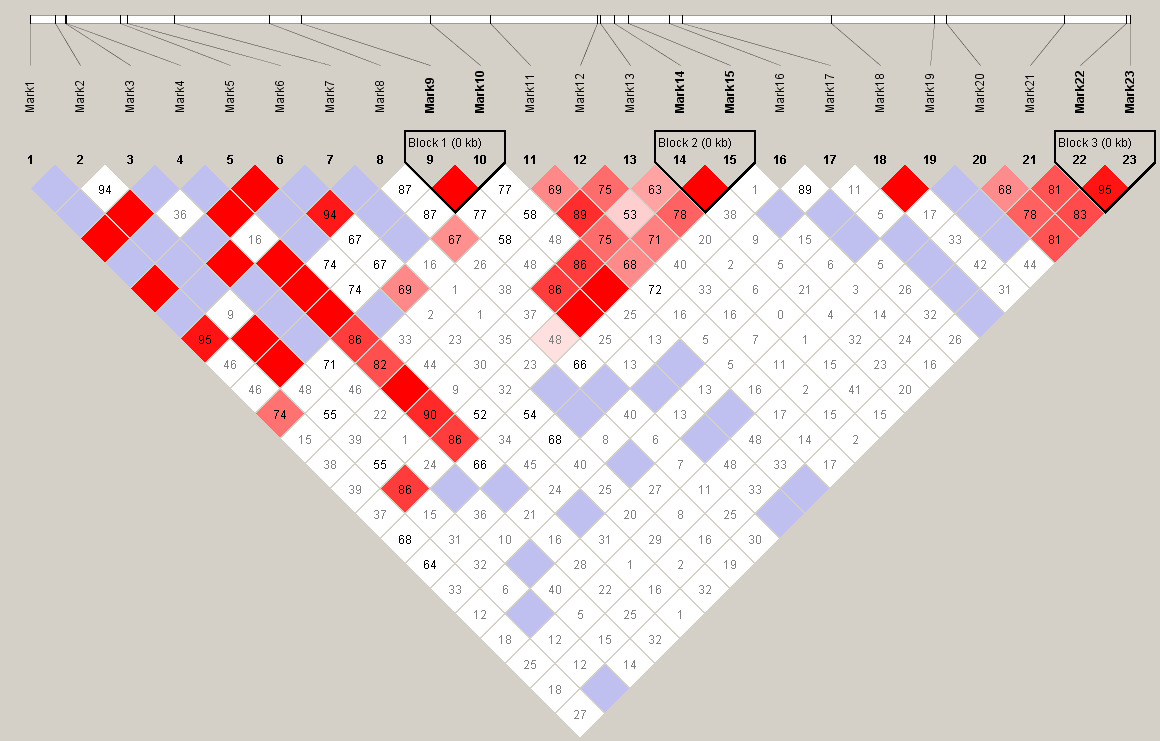


Supplemental Figure 1: Linkage disequilibrium (LD) of IL-32 in the Caucasian population.

Supplemental figure 2: Case-control probability of developing infection associated ALI

Supplemental figure 3: Death at day 60

Supplemental figure 4: Shock as defined by Brussels score criteria

Supplemental figure 5: Shock as defined by sequential measurement of vital signs

Supplemental figure 6: Shock as determined by sequential measurement of vital signs.

| Haplotype | Freq. in total^a^ | Freq. in control^b^ | Freq. in case | | |
| --- | --- | --- | --- | --- | --- |
|  |  |  | Total^c^ | No Bruss^d^ | Bruss^e^ |
| GGCAAAC | 0.018 | 0.016 | 0.015 | 0 | 0.018 |
| GGCAAAT | 0.022 | 0.024 | 0.023 | 0 | 0.027 |
| GGCAAGT | 0.031 | 0.031 | 0.034 | 0 | 0.040 |
| GGCATAC | 0.038 | 0.050 | 0.041 | 0.043 | 0.034 |
| GGCATGT | 0.014 | 0.012 | 0.005 | 0.018 | 0.005 |
| GGCGTAC | 0.024 | 0.014 | 0.032 | 0.024 | 0.034 |
| GTCAAAC | 0.022 | 0.018 | 0.023 | 0 | 0.016 |
| GTCAAGT | 0.014 | 0.013 | 0.022 | 0 | 0.027 |
| GTCATAC | 0.027 | 0.018 | 0.037 | 0.134 | 0.029 |
| GTCATAT | 0.014 | 0.010 | 0.018 | 0.035 | 0.009 |
| GTCATGT | 0.014 | 0.013 | 0.011 | 0 | 0.012 |
| TGCATAC | 0.047 | 0.051 | 0.038 | 0.064 | 0.029 |
| TGCATGT | 0.029 | 0.035 | 0.025 | 0 | 0.035 |
| TGCGTAC | 0.034 | 0.024 | 0.037 | 0.076 | 0.038 |
| TGCGTAT | 0.016 | 0.015 | 0.022 | 0 | 0.029 |
| TTCAAGT | 0.014 | 0.009 | 0.018 | 0.017 | 0.012 |
| TTCATAC | 0.031 | 0.027 | 0.036 | 0.034 | 0.038 |
| TTCGTAC | 0.019 | 0.025 | 0.014 | 0 | 0.013 |
| TTTAAAC | 0.059 | 0.077 | 0.046 | 0.088 | 0.039 |
| TTTAAAT | 0.012 | 0.009 | 0.010 | 0.051 | 0 |
| TTTAAGT | 0.052 | 0.047 | 0.066 | 0.072 | 0.068 |
| TTTATAC | 0.146 | 0.160 | 0.132 | 0.107 | 0.139 |
| TTTATAT | 0.011 | 0.014 | 0.008 | 0.000 | 0.007 |
| TTTATGT | 0.055 | 0.060 | 0.055 | 0.044 | 0.060 |
| TTTGTAC | 0.017 | 0.010 | 0.027 | 0.017 | 0.035 |
| TTTGTGC | 0.018 | 0.014 | 0.020 | 0 | 0.022 |
| TTTGTGT | 0.011 | 0.010 | 0.012 | 0 | 0.010 |

^a^ Haplotype frequencies in the entire sample

^b^ Haplotype frequencies in control group

^c^ Haplotype frequencies in case group

^d^ Haplotype frequencies in the group of patients without Bruss

^e^ Haplotype frequencies in the group of patients with Bruss

Supplemental Table 2: Haplotypes in the region 1474-4153 with frequencies larger than 1%
